# Interictal Discharge Pattern in Preschool-Aged Children With Tuberous Sclerosis Complex Before and After Resective Epilepsy Surgery

**DOI:** 10.3389/fneur.2022.868633

**Published:** 2022-05-31

**Authors:** Liu Yuan, Yangshuo Wang, Shuhua Cheng, Junchen Zhang, Shaohui Zhang, Tinghong Liu, Guojun Zhang, Shuli Liang

**Affiliations:** ^1^Functional Neurosurgery Department, National Children's Health Center of China, Beijing Children's Hospital, Capital Medical University, Beijing, China; ^2^Key Laboratory of Major Diseases in Children, Ministry of Education, Beijing, China; ^3^Neurology Department, National Children's Health Center of China, Beijing Children's Hospital, Capital Medical University, Beijing, China; ^4^Neurosurgery Department, Affiliated, Jining Medical College, Jining, China; ^5^Neurosurgery Department, People's Liberation Army (PLA) General Hospital, Beijing, China

**Keywords:** epilepsy, epileptogenic tuber, interictal discharge (IID), scalp electroencephalographs (EEGs), tuberous sclerosis complex (TSC)

## Abstract

**Objective:**

To analyze the interictal discharge (IID) patterns on pre-operative scalp electroencephalogram (EEG) and compare the changes in IID patterns after removal of epileptogenic tubers in preschool children with tuberous sclerosis complex (TSC)-related epilepsy.

**Methods:**

Thirty-five preschool children who underwent resective surgery for TSC-related epilepsy were enrolled retrospectively, and their EEG data collected before surgery to 3 years after surgery were analyzed.

**Results:**

Twenty-three (65.7%) patients were seizure-free post-operatively at 1-year follow-up, and 37–40% of post-operative patients rendered non-IID on scalp EEGs, and patients with focal IIDs or generalized IID patterns on pre-operative EEG presented a high percentage of normal post-operative scalp EEGs. IID patterns on pre-operative scalp EEGs did not influence the outcomes of post-operative seizure controls, while patients with non-IID and focal IID on post-operative EEGs were likely to achieve post-operative seizure freedom. Patients with new focal IIDs presented a significantly lower percentage of seizure freedom than those without new focal IIDs on post-operative EEGs at 3-year follow-up.

**Conclusion:**

Over 1/3 children with TSC presented normal scalp EEGs after resective epileptsy surgery. Patients with post-operative seizure freedom were more likely to have non-IIDs on post-operative EEGs. New focal IIDs were negative factors for seizure freedom at the 3-year follow-up.

## Highlights

- The pre-operative interictal discharge patterns on scalp EEGs had no obvious effect on post-operative seizure control in TSC-related epilepsy.
- The interictal discharge patterns on post-operative scalp EEGs were consistent in TSC patients who underwent resective epileptic surgery.
- In 37–40% of post-operative patients, interictal discharge on scalp EEGs were not present.
- Patients with post-operative seizure freedom were more likely to have absent interictal discharge on post-operative scalp EEG.

## Introduction

Approximately 90% of patients with tuberous sclerosis complex (TSC) suffer from epilepsy, which are often medically resistant and can present with multiple seizure types (1–3). Epilepsy surgery, especially resective surgery, is the most efficient approach for patients with TSC-related intractable epilepsy, and 68–75% of patients with TSC present post-operative seizure freedom and a worthwhile reduction (>90%) in seizure frequency after resective surgery (4–7).

Due to an autosomal dominant epilepsy with multiple tubers in most patients with TSC-related epilepsy, the biggest questions to the resective surgery for TSC are whether the numbers and volumes of cortical tubers, and the number of epileptogenic tubers will increase with age, and whether new epileptogenic tubers will appear after removal of epileptogenic tubers, besides the difficulty in localizing the epileptogenic tuber(s). It has been reported that the number and relative volume of cortical tubers will not change after 1 year of age in TSC patients (8), and consistent location of interictal epileptiform activity on scalp electroencephalographs (EEGs) indicated the relative stability of epileptogenic tubers in patients with TSC-related epilepsy (9).

As previously reported, total resection of the actual seizure-onset zone does not always lead to seizure freedom in focal seizures. In some cases, additional post-surgical recordings suggest that areas adjacent to the resection trigger epileptic seizures. These observations led to the concept of potential seizure-onset zones (10). Therefore, surgical resections did not result in seizure freedom either because of incomplete resection of the actual seizure-onset zone or incomplete resection of the potential seizure-onset zone (10). Weiner et al. reported a three-stage operation in patients with TSC-related epilepsy (11). They conserved subdural intracranial electrodes for several days after the first resective surgery and found that the margins of the resection and other tubers could induce seizures. However, there is no long-term study on whether new epileptogenic tubers will present and provoke seizures after resective surgery. Hence, the long-term IID patterns on post-operative EEG were analyzed to study whether there were new epileptogenic tubers after the removal of epileptogenic tubers.

## Methods

### Patients

Patients were enrolled retrospectively according to the following criteria: patients who were no more than 6-year-old; subjects who underwent resective surgery from January 2015 to June 2020 in the comprehensive epilepsy centers of our hospitals; subjects who had been diagnosed with TSC according to the revised diagnostic criteria of Northrup et al. (12); and patients who had pre-operative scalp EEG recordings and post-operative scalp EEG recording at 2-year follow-up. The study was approved by the ethics committee of the Fourth Medical Center of the PLA General Hospital (Permit No. 2019KY004-HS001).

### Pre-operative Evaluation

Non-invasive pre-operative evaluations included neurological physical examinations, high-resolution magnetic resonance imaging (MRI), long-term video EEG recordings, interictal positron emission tomography (PET), and Gesell development quotient tests. MRI scans included 3.0T routine axial T1- and T2-weighted, diffusion-weighted, and sagittal T1-weighted imaging and 2-mm thickness/zero interval axial and coronal T2-flair imaging. Each scalp EEG recording had to include more than three habitual seizures. MRI-PET co-registration was performed for each patient. Stereo-EEGs were recorded to detect epileptogenic cortex tubers when the predominant cortex tuber on MRI or the areas with focal ictal symptoms were discrepancies with the region with the focal scalp EEG (7, 13). The epileptogenic tuber was defined as the first tuber with initial rhythmic discharge on scalp EEG or stereo-EEG before a clinical seizure attack.

### Pre-operative and Post-operative Scalp EEG Recording and Analysis

**Figure 1:**
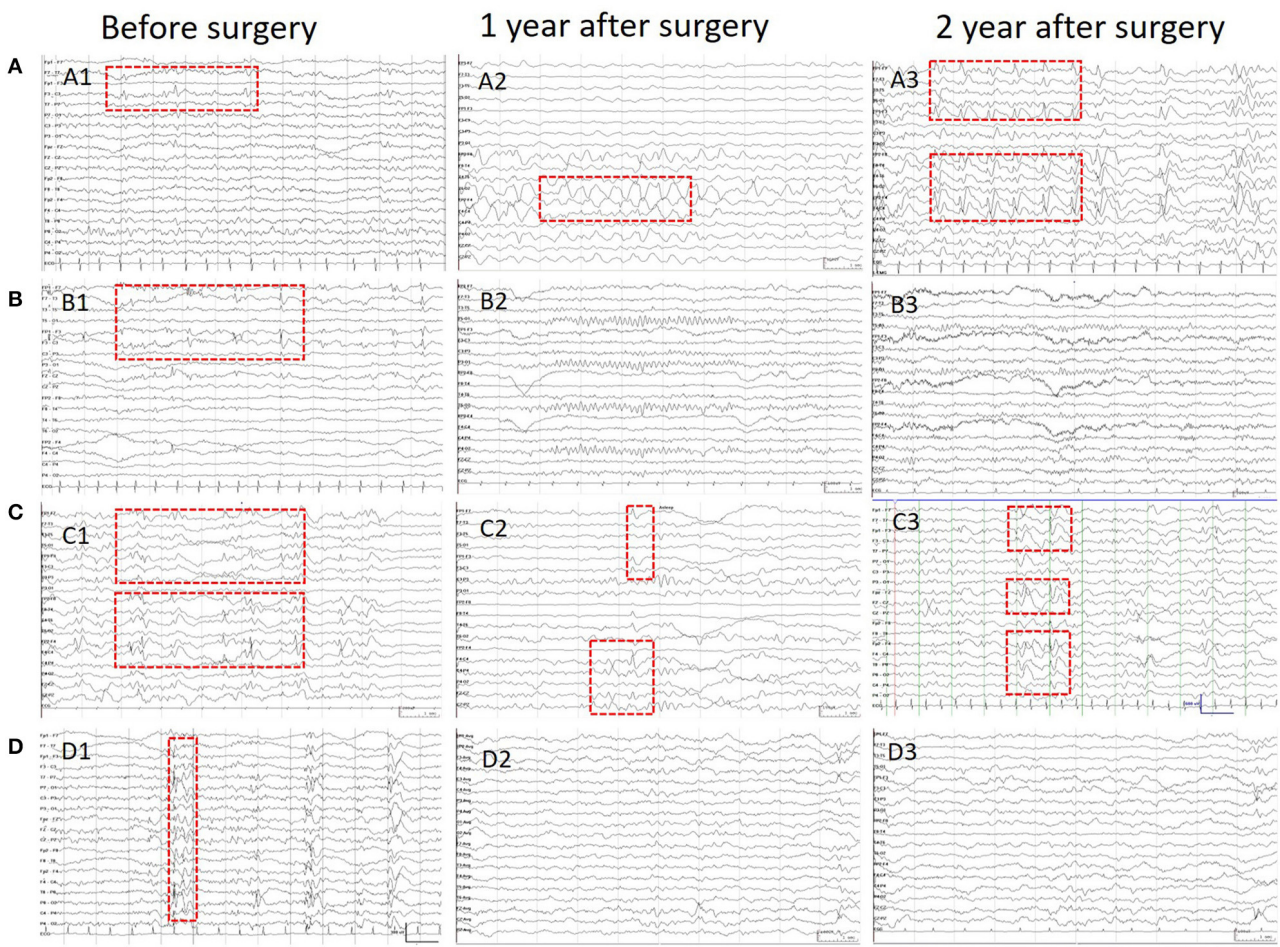
Patients' IIDs patterns on EEG before and after surgeries. This figure shows the different IID patterns of four cases on pre-operative and post-operative scalp EEGs. **(A)** Shows the focal IIDs on the left frontal areas on pre-operative EEG (A1), focal IIDs and slow waves on the right frontal areas on EEG in 1 year after surgery (A2), and multifocal IIDs on the EEG in 2 years after surgery. **(B)** Shows the focal IIDs on pre-operative EEGs (B1), and non-IIDs on EEGs in 1- (B2) and 2-year after surgery (B3). **(C)** Shows the multifocal IIDs on pre-operative EEGs (C1), and multifocal IIDs on EEGs in 1- (C2) and 2-year (C3) after surgery. **(D)** Shows the generalized IIDs on pre-operative EEGs (D1), and non-IIDs on EEGs in 1- (D2) and 2- year (D3) after surgery.

**Figure 2:**
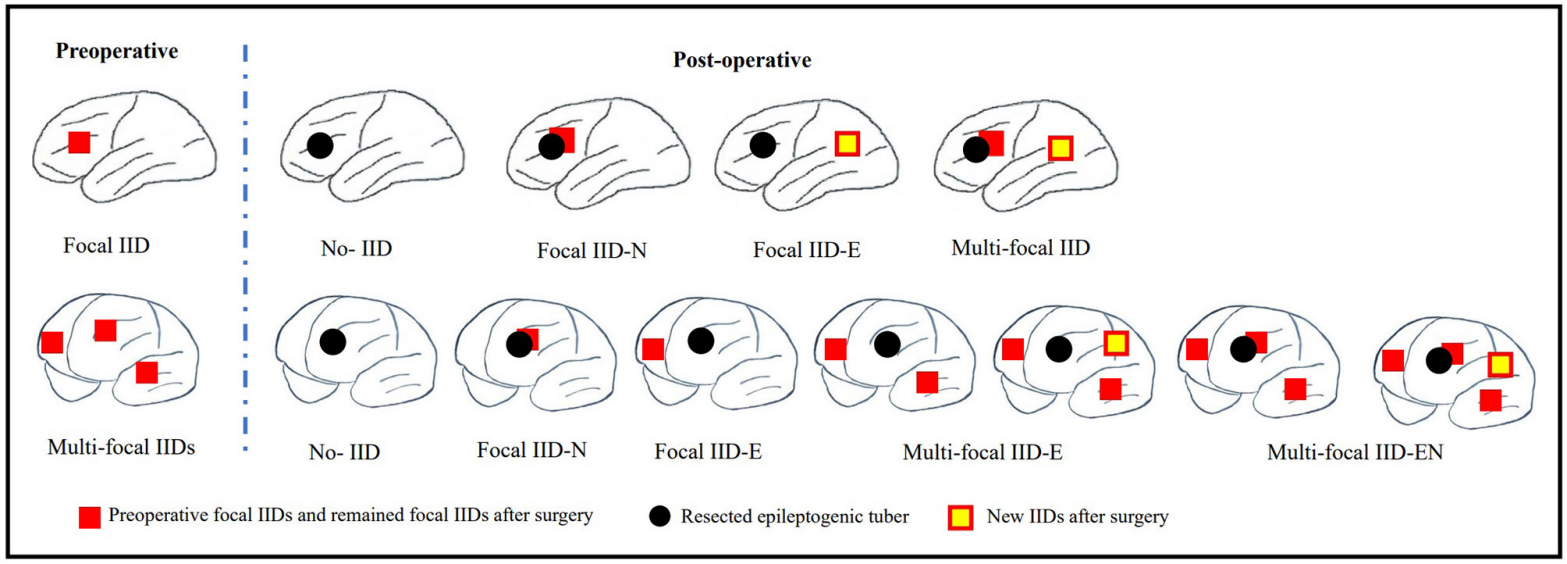
Schematic diagram of pre-operative focal IID and multi-focal IID patterns and their post-operative IID patterns. The top row of this figure shows the pre-operative focal IID pattern and its post-operative patterns, including non-IID, focal IID-N (continuous IID located near the resected region), focal IID-E (new IID located elsewhere to the resected region), and multifocal IID-EN (new IID located elsewhere to the resected region and continuous IID located near the resected region). The bottom row of this figure shows the pre-operative multiple focal IID pattern and its post-operative patterns, including non-IID, focal IID-N, focal IID-E (continuous IID located elsewhere to resected region), multifocal IID-E (continuous IID or continuous IID and new IID located elsewhere to regions with pre-operative IIDs), multi-focal IID-EN (continuous IID or new IID located elsewhere to regions with pre-operative IID, and continuous IID located near the resected region).

Scalp EEGs were made with 21-channel recordings with electrode positions according to the 10–20 system. Digital recordings of EEG traces were available for assessment. All scalp EEGs were recorded for no <24 h. Two observers (L.Y and S.C) reviewed all the EEGs. Both observers were blinded to the previous reports and recording time of EEG, information about patients' name and seizure semiology or frequency, and MRI findings. A final consensus reading was performed to identify the location of the epileptiform abnormalities. Consistency was defined as the presence of interictal epileptiform activity at the same location in all EEGs reviewed. If the assessment did not correspond to the original assessment, a third observer (S.L) reviewed the recordings. The results of interictal EEG were divided into four groups: non-interictal discharge (IID) (without interictal epileptiform discharges, IIDs) (Figure 1B,B3), focal IID (unique unilateral focal IID with or without generalized IID) (Figures 1A,A1,A2, 2), multifocal IID (two or more independent focal IID or unilateral IID with or without generalized IID) (Figures 1C,C1,C2, 2), and generalized IID (any bilaterally synchronous and symmetric pattern IID without focal IIDs, but it can be in a restricted field) (14) (Figure 1D,D1,D2). For the post-operative interictal EEG patterns, focal/multifocal IID was subdivided into focal/multi-focal IID-E (new IID located elsewhere to region with pre-operative IIDs) a focal/multi-focal IID-N (continuous IID located near the resected region), multi-focal IID-EN (new IID located elsewhere to region with pre-operative IIDs and continuous IID located near the resected region), multi-focal/focal IID (focal/multi-focal IID located regions with pre-operative multiple focal IID but the resected region), and non-IID (Figure 2).

### Surgical Methods and Post-operative Medicine Treatment

The patient underwent lobectomy and/or tuberectomy. Tuberectomy was used for the epileptogenic tuber within or near the eloquent area. Lobectomy was performed for large epileptogenic tubers or multiple epileptogenic tubers in the anterior temporal lobe, frontal pole, or occipital pole. Multiple tuberectomies or lobectomy combined with tuberectomy were considered when multiple epileptogenic tubers or epileptogenic and propagating tubers could not be removed by a single lobectomy. Pre-operative and post-operative medicine treatment was provided to all patients with 2–4 types of optimal anti-seizure medications.

### Statistical Analysis

Statistical analyses were performed using SPSS software (version 26.0; SPSS, Inc., Chicago, IL, USA). The outcomes were described as percentages, means, and standard deviations. McNemar-Bowker's test was used to analyze influence of the IIDs patterns on pre-operative EEG on the outcome of IIDs patterns on post-operative EEG. Generalized estimating equation (GEE) was used to analyze the influences factors on the post-operative seizure control of the three time points of follow-up. Chi-square and Fisher's exact tests were performed for univariate analyses of categorical variables. When the two-tailed error probability “*p*” was < 0.05, the outcome was considered significant.

## Results

### Patients

Fifty-three preschool children (0–6 years old) with TSC-related intractable epilepsy underwent epilepsy surgery in our hospital from January 2015 to December 2019, and 35 of them, who had pre-operative and post-operative EEG recordings and surgical outcomes, were enrolled in this retrospective study. There were 11 (31.4%) girls and 24 (68.6%) boys. The average age at surgery was 3.51 [SD = 1.69, range (0.7–6.0), medium: 3.0, interquartile range (2.0–5.0)] years, the average age at first unprovoked seizure was 0.90 [SD = 1.13, range (0.0–5.0), medium 0.6, interquartile range (0.3–1.0)] years, and the pre-operative history of seizure ranged from 0.7 to 5.7 [mean = 2.55, SD = 1.50, medium 2.2, interquartile range (1.2–3.5)] years. Stereo-EEGs were performed on 11 (31.4%) of the 35 children.

### Surgery Approach and Post-operative Seizure Freedom

The surgical approach consisted of 15 tuberectomies, 9 lobectomies, and 11 multiple tuberectomies or lobectomies combined with tuberectomies. All 35 patients completed 1- and 2-year follow-up, and 25 (71.4%) and 23 (65.7%) of them had post-operative seizure freedom. Eighteen (60%) out of the 30 patients who completed the 3-year follow-up had post-operative seizure freedom.

### Pre-operative and Post-operative IIDs

**Figure 3:**
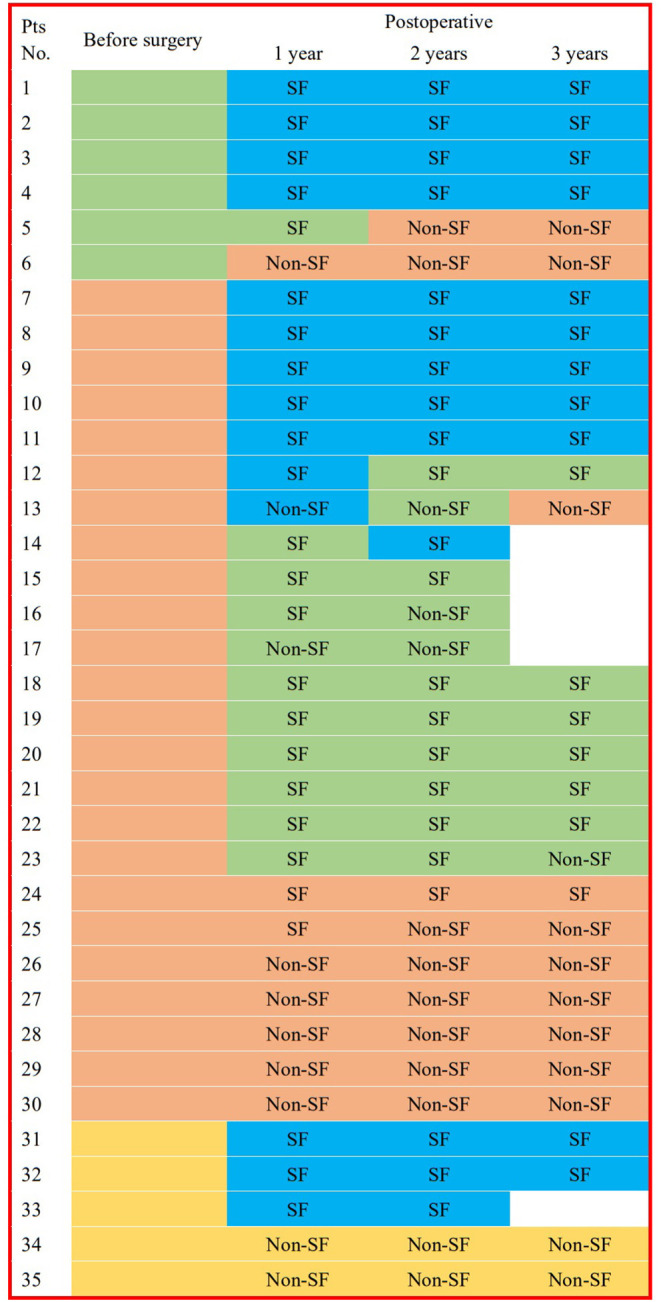
IID patterns on scalp EEGs in different times for each patient. This figure shows the interictal epileptiform discharge (IID) patterns from 3 years before the surgery to 3 years after surgery in every patient. Blue box for EEG without IID; green box for focal IIDs; orange box for multiple focal IIDs, and golden box for generalized IIDs. pts, patients; pre-op, pre-operative; post-operative, post-operative; SF, seizure freedom.

All pre-operative scalp EEGs indicated IIDs. All patients had scalp EEGs 1-year (40% without IID) and 2 years (37.1% without IID) after surgery, respectively, and 30 (36.7% without IID) children had post-operative scalp EEGs at the 3-year follow-up. No significant difference was found in the percentage of non-IID on scalp EEGs in the different post-operative periods (*p* = 0.8929). Eleven patients did not present with IIDs on post-operative scalp EEGs at all three follow-ups, and one case (3%) with focal IID on scalp EEG at 1-year follow-up reached normal EEG at 2-year follow-up, and two patients with normal EEGs at 1-year follow-up presented with focal or multi-focal IIDs at 2- and 3-year follow-up (Figure 3). There were significant differences in the outcomes of IID patterns on post-operative scalp EEGs at 1- (*p* = 0.0016), 2- (*p* = 0.0038) or 3-year (*p* = 0.0322) follow-up among patients with different IID patterns on pre-operative scalp EEG (Table 1; Figure 3).

**Table 1:** Relationships between IIDs patterns on pre- and post-operative EEGs.

**Post-operative IID in EEG**	**Pre-operative IID in EEG**			**Chi-square**	***P-*value**
	**Focal IIDs**	**Multiple IIDs**	**Generalized IIDs**		
**1 year follow-up**					
No IID	4 (67%)	7 (29%)	3 (60%)	21.36	0.0016
Focal IID	1 (17%)	10 (42%)	0 (0%)		
Multiple IIDs	1 (17%)	7 (29%)	0 (0%)		
Generalized IIDs	0 (0%)	0 (0%)	2 (40%)		
**2 years follow-up**					
No IID	4 (67%)	6 (25%)	3 (60%)	19.23	0.0038
Focal IID	0 (0%)	11 (46%)	0 (00%)		
Multiple IIDs	2 (33%)	7 (29%)	0 (0%)		
Generalized IIDs	0 (0%)	0 (0%)	2 (40%)		
**3 years follow-up**					
No IID	4 (67%)	5 (25%)	2 (50%)	13.78	0.0322
Focal IID	0 (0%)	7 (35%)	0 (0%)		
Multiple IIDs	2 (33%)	8 (40%)	0 (0%)		
Generalized IIDs	0 (0%)	0 (0%)	2 (50%)		

*IIDs, interictal epileptiform discharges*.

### Patients' Characteristics and Post-operative Seizure Freedom

The influence of age at surgery, age at first seizure, seizure type at onset, pre-operative history, and number of resected tubers on the post-operative seizure control was not found. However, patients with pre-operative seizure history <2 years presented statistically significant higher percentage of post-operative of seizure free than those with seizure history longer than 2 years (OR = 6.387, 95%CI: 1.174–34.746, *p* = 0.032) (Table 2).

**Table 2:** Relationship of pre-operative influence factors and number of resected tuber and post-operative seizure freedom.

**Factors**	**Number (%) of seizure freedom**			**OR**	**95%CI**	***P-*value**
	**1-year FU**	**2-year FU**	**3-year FU**			
**Age at surgery**						
<3 years	12 (71%)	11 (65%)	9 (64%)	1.737	0.709–4.254	0.227
≧3 years	13 (72%)	11 (61%)	9 (56%)	-	-	-
**Age at first seizure**						
<1 year	18 (72%)	15 (60%)	12 (55%)	2.500	0.635–9.841	0.190
≧1 years	7 (70%)	7 (70%)	6 (75%)	-	-	-
**Seizure type at onset**						
Focal seizure	12 (80%)	10 (67%)	8 (62%)	1.154	0.158–8.415	0.888
Generalized spasm	9 (64%)	8 (57%)	6 (55%)	0.719	0.100–5.187	0.743
Other seizures	4 (67%)	4 (67%)	4 (67%)	-	-	-
**History of pre**-operative seizure^*^						
<2 years	13 (87%)	13 (87%)	11 (85%)	6.387	1.174–34.746	0.032
≧2 years	12 (60%)	9 (45%)	7 (41%)	-	-	-
**Number of resected tubers**						
1	14 (70%)	12 (60%)	11 (65%)	1.850	0.101–33.942	0.679
2	10 (77%)	9 (69%)	6 (55%)	2.083	0.106–40.780	0.629
3	1 (50%)	1 (50.00)	1 (50%)	-	-	-
**Pre**-**operative EEG pattern**						
Focal IIDs	5 (83%)	4 (67%)	4 (67%)	1.950	0.174–21.871	0.588
Multiple IIDs	17 (74%)	16 (67%)	12 (60%)	1.375	0.192–9.842	0.751
Generalized IIDs	3 (60%)	3 (60%)	2 (50%)	-	-	-

*IIDs, interictal epileptiform discharges; FU, follow-up*.

* *P < 0.05 with GEE analysis*.

### IIDs Patterns and Post-operative Seizure Freedom

IID patterns on scalp EEGs before surgery did not affect the outcomes of post-operative seizure control (*p* > 0.05, Table 2). Significant differences were found in patients' seizure controls at 1-, 2-, and 3-year follow-up among patients with different IID patterns on scalp EEGs at the same time after surgery (*p* < 0.001, Table 3). Non-IIDs and focal IIDs on post-operative EEGs indicated the highest percentage of seizure freedom (Table 3).

**Table 3:** Influence of post-operative EEG patterns on post-operative seizure freedom.

**IIDs in EEG**	**1-year follow-up**		**2-year follow-up**		**3-year follow-up**	
	**SF**	***P-*value**	**SF**	***P-*value**	**SF**	***P-*value**
EEG pattern at follow up^*^						
Non-IID	13 (93%)	<0.001	13 (100%)	<0.001	11 (100%)	<0.001
Focal IIDs	10 (91%)		8 (73%)		6 (86%)	
Multiple IIDs	2 (25%)^*^		1 (11%)^*^^#^		1 (10%)^*^^#^	
Generalized IIDs	0 (0%)^*^^#^		0 (0%)^*^^#^		0 (0%)^*^^#^	
**Post-operative continuous IID located near the resected region**						
Yes	5 (45%)	0.421	6 (46%)	0.157	5 (45%)	0.266
No	20 (83%)		17 (77%)		13 (68%)	
**New IID located elsewhere to the resected region on post-operative EEG compared to pre-operative EEG**						
Yes	0 (0%)	0.076	1 (25%)	0.134	0 (0%)	0.018^$^
No	25 (76%)		21 (68%)		18 (69%)	

*IIDs, interictal epileptiform discharges; SF, seizure freedom*.

* *P < 0.05, the SF percentage in the group compared to the data in non-IID group at same follow-up*.

# *P < 0.05, the SF percentage in the group compared to the data in focal-IID group at same follow-up*.

$ *P < 0.05, the SF percentage in the group compared to the data in other group at same follow-up*.

### IIDs in Areas of Resected Tubers and New Finding of Focal IIDs After Surgery

There were 21 patients who underwent a single area resection (six lobectomies and 15 tuberectomies), and 14 patients underwent multiple area resections (three lobectomies combined tuberectomies, and 11 multiple tuberectomies). In the resected areas, 25 (71.4%), 22 (62.8%), and 19 (63.3%) patients' scalp EEGs did not have IIDs in the resected areas at 1-, 2-, and 3-year follow-up, respectively (Table 3), and no significant difference was found among the percentages of patients without IIDs in the resected area among the three follow-ups (Table 3). No significant difference was found in post-operative seizure control between patients with IIDs in the resected area and those without IIDs in the resected area (Table 3).

Compared to all pre-operative scalp EEG results before surgery in each patient, there were two (5.7%), four (11.4%), and four (13.3%) patients with new focal IIDs on scalp EEGs at 1–3 years of follow-ups, respectively, and there was no significant difference in the percentage of new focal IIDs among the three follow-ups (*p* = 0.5588). However, significant difference was found in patients' seizure controls at 3-year follow-up between patients with or without new focal IID on post-operative EEGs (*p* = 0.018, Table 3).

## Discussion

To the best of our knowledge, this is the first study to examine post-operative scalp EEGs in patients with TSC-related epilepsy after resective surgery. Although ictal scalp EEG and intracranial EEG have been used to localize the onset zone of seizures, interictal EEG is usually used to define the irritation area (2, 6, 7, 15, 16). However, some studies have confirmed that scalp EEG can also be a biomarker significantly associated with an underlying cortical pathology and aid in localizing suspicious brain regions (2, 17–20). Furthermore, interictal EEG patterns have been reported to be associated with post-operative seizure control in patients with TSC-related epilepsy (4, 21). Because over 70% of patients reach post-operative seizure freedom at the 1-year follow-up and 2–4 h of scalp video-EEG is routinely used for post-operative EEG examination, ictal EEG cannot be recorded in most patients at follow-up. Moreover, intracranial EEG is not suitable for post-operative follow-up. Therefore, interictal epileptiform discharge on scalp EEG is one of the best tools to observe long-term EEG changes after removal of the epileptogenic tubers in TSC patients.

The outcomes in this cohort show that patients with multifocal or generalized IIDs, besides focal IIDs, on pre-operative scalp EEG can also present non-IIDs on post-operative EEGs at 1–3 years of follow-up, which proves that focal epileptogenic tubers could lead to multifocal or generalized IIDs. Furthermore, patients with pre-operative multifocal or generalized IIDs do not have a low percentage of post-operative seizure freedom compared to those with pre-operative focal IID, which indicates that pre-operative IID patterns have no obvious effect on post-operative seizure control in carefully selected patients and comprehensive pre-operative evaluations. Due to the lower myelinization of the brain and less organized brain networks (22), young children with focal onset zone can presence diffuse interictal activity pre-operatively.

The maximal brain growth rate occurs around birth, and the brain is ~95% of the size of the adult brain by 6 years of age. The bulk of this early growth comes from a variety of sources, including increases in synapses and dendrites, as well as myelination (23). Therefore, it may be the most obvious period of EEG discharge pattern changes. Nevertheless, 6–12% of patients presented new focal IIDs in 1–3 years of follow-up after removal of epileptogenic tubers compared to pre-operative IID patterns on scalp EEG, and 37–40% patients did not have obvious IIDs on post-operative scalp EEG, including 11 patients without IID for 3 years. Furthermore, obvious correlations in IIDs patterns in post-operative EEG were found among 1-, 2- and 3-year follow-up, which indicated stability of IIDs patterns in post-operative EEG. Concurrently, 60% of post-operative patients achieved seizure freedom for 3 years. Therefore, we first showed that the epileptogenic tubers in patients with TSC-related epilepsy were relatively stable, and reconfirmed that not all tubers were independent epileptogenic tubers.

Continuous IIDs near the resected tubers can also be found in 29–37% of scalp EEGs in post-operative patients. The reasons for the continuous IID may include incomplete removal of tubers, propagation from IID in other tubers, and hyperexcitability of the cortex near the epileptogenic tubers. However, the presence of continuous IID did not affect the post-operative seizure freedom, which indicated that the incomplete removal of tubers should not be the main reason.

This study has some limitations. First, not all patients had all the 3-year EEG results before and after surgery due to the limitations of a retrospective study. Second, the sample of enrolled subjects was not large because of the low incidence of TSC-related epilepsy and the limited number of patients who underwent resective surgery.

In conclusion, pre-operative IID patterns had no obvious effect on post-operative seizure control after comprehensive pre-operative evaluations, while patients with focal IIDs or generalized IID patterns on pre-operative EEG presented a high percentage of normal post-operative scalp EEGs. In 37–40% of post-operative patients, non-IID on scalp EEGs was shown, and patients with post-operative seizure freedom were more likely to have non-IID on post-operative EEGs. New focal IIDs were negative factors for seizure freedom at the 3-year follow-up. The IID patterns on post-operative scalp EEGs were consistent, and epileptogenic tubers in patients with TSC-related epilepsy were relatively stable.

## Data Availability Statement

The raw data supporting the conclusions of this article will be made available by the authors, without undue reservation.

## Ethics Statement

The studies involving human participants were reviewed and approved by the Fourth Medical Center of the PLA General Hospital. Written informed consent from the participants' legal guardian/next of kin was not required to participate in this study in accordance with the national legislation and the institutional requirements.

## Author Contributions

SL: conceptualization, methodology, writing-original draft preparation, writing-reviewing and editing, funding acquisition, and investigation. LY and YW: methodology, writing-original draft preparation, and investigation. SZ and JZ: subjects collections and investigation. TL: investigation and data analysis. SC and GZ: supervision and writing-reviewing and editing. All authors contributed to the article and approved the submitted version.

## Funding

This research was supported by National Nature and Science Foundation of China (82071448, SL) and Beijing Nature and Science Foundation of China (7202045, SL).

## Conflict of Interest

The authors declare that the research was conducted in the absence of any commercial or financial relationships that could be construed as a potential conflict of interest.

## Publisher's Note

All claims expressed in this article are solely those of the authors and do not necessarily represent those of their affiliated organizations, or those of the publisher, the editors and the reviewers. Any product that may be evaluated in this article, or claim that may be made by its manufacturer, is not guaranteed or endorsed by the publisher.
