# Supplementary figures and images for: Interictal Discharge Pattern in Preschool-Aged Children With Tuberous Sclerosis Complex Before and After Resective Epilepsy Surgery
Source: Front Neurol. 2022 May 31;13:868633. doi: 10.3389/fneur.2022.868633 (PMC9197454; doi:10.3389/fneur.2022.868633)

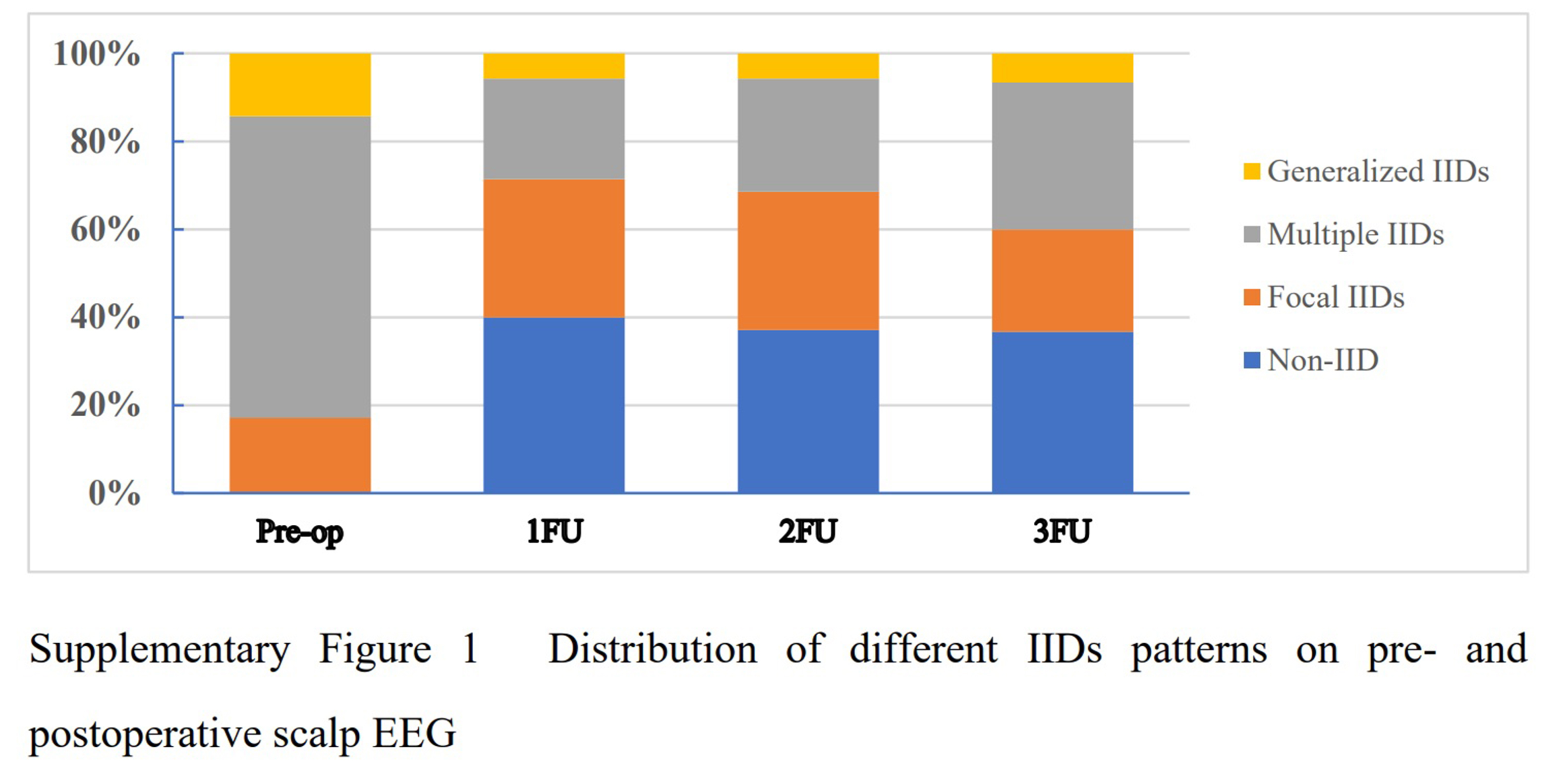

Supplement: Supplementary file 1 [file Image_1.JPEG]
